# Supplementary material for: Clinical Significance of COVID-19 and Diabetes: In the Pandemic Situation of SARS-CoV-2 Variants including Omicron (B.1.1.529)
Source: Biology (Basel). 2022 Mar 4;11(3):400. doi: 10.3390/biology11030400 (PMC8945151; doi:10.3390/biology11030400)
Supplement: Supplementary file 1 [file biology-11-00400-s001.zip › biology-1521707-supplementary.pdf]

**Table S1.** Overview of the risk for COVID-19-related mortality according to the glycemic control status before hospitalization.

| Author | Study Population | Region | Period | No. of Patients | HbA1c level mmol/mol | HbA1c level % | Outcome | Risk | | Ref. No. |
|---|---|---|---|---|---|---|---|---|---|---|
| Holman et al. | Nationwide population-based cohort study | United Kingdom | March 1–May 6 2020 | T1D 264,390, T2D 2,874,020, COVID-19-related deaths: T1D 464, T2D 10,525 | 48–53 | 6.5–7.0 | Mortality | ref | | [26] |
| | | | | | 59–74 | 7.6–8.9 | | T1D: HR 1.16(0.81–1.67), T2D: HR 1.22(1.15–1.30) | * | |
| | | | | | 75–85 | 9.0–9.9 | | T1D: HR 1.37(0.90–2.07), T2D: HR 1.36(1.24–1.50) | * | |
| | | | | | ≥86 | ≥ 10 | | T1D: HR 2.23(1.50–3.30), T2D: HR 1.61(1.47–1.77) | * | |
| Cariou et al. | Multi-center cohort study | France | March 10–March 31 2020 | 846 HbA1c available, total: 1,317, T1D 39, T2D 1,166 | <3 | <7.0 | Mortality | ref | | [27] |
| | | | | | 53–63 | 7.0–7.9 | | OR 1.55(0.82–2.93) | ** | |
| | | | | | 64–74 | 8.0–8.9 | | OR 1.09(0.52–2.28) | ** | |
| | | | | | ≥75 | ≥9 | | OR 0.84(0.40–1.75) | ** | |
| Williamson et al. | Nationwide population-based cohort study | United Kingdom | January 1–May 6 2020 | Total 17,278,392, COVID-19-related deaths 10,926 | < 58 | <7.5 | Mortality | HR: 1.31(1.24–1.37) | # | [32] |
| | | | | | ≥58 | ≥7.5 | | HR: 1.95(1.83–2.08) | # | |

T1D: type 1 diabetes, T2D: type 2 diabetes, HR: hazard ratio, OR: odds ratio. * adjusted for sex, age, ethnicity, socioeconomic deprivation, renal impairment, BMI (body mass index), tobacco smoking status, and cardiovascular comorbidities. ** adjusted for sex and age. # adjusted for age, sex, BMI, smoking, IMD (index of multiple deprivation) quintile, and comorbiditiessevere asthma, respiratory disease, chronic heart disease, liver disease, stroke, dementia, other neurological diseases, reduced kidney function, autoimmune diseases (rheumatoid arthritis, lupus or psoriasis) and other immunosuppressive conditions.

**Table S2.** Overview of the risk for adverse clinical outcomes of COVID-19 according to the blood glucose levels at admission.

| Author | Study Population | | Region | Period | No. of Patients | Parameter of Hyperglycemia | | Outcome | Risk | Ref. No. |
|---|---|---|---|---|---|---|---|---|---|---|
| Cariou et al. | Multi-center cohort study | Diabetes | France | March 10–31 2020 | 940 admission plasma glucose available, total 1,317 | PG | > 5.55 mmol/L | Tracheal intubation and/or death within 7 days of admission<br>7-day mortality | OR 1.28 (1.12–1.48) [a]<br>OR 1.20 (0.98–1.46) [a] | [27] |
| Wang et al. | Multi-center retrospective cohort study | Hospitalized patients without a previous diagnosed diabetes | China | January 24–February 10 2020 | 605 | FBG | ≥ 7.0 mmol/L | Mortality | HR 2.30(1.49–3.55) | [34] |
| | | | | | | FBG | < 6.0 mmol/L<br>6.1–6.9 mmol/L<br>≥ 7.0 mmol/L | In hospital complications * | ref<br>OR: 2.61(1.64–4.41)<br>OR: 3.99(2.71–5.88) | |
| Yang et al. | Single-center, retrospective study | Hospitalized | China | February 8– March 10 2020 | 263 | FPG | 7.0–11.0 mmol/L<br>≥ 11.1 mmol/L | Mortality | HR 1.90(1.11–3.25) [b]<br>HR 2.09(1.21–3.64) | [35] |
| Zhang et al. | Multi-center, retrospective cohort study | Hospitalized | China | January 1–March 17 2020 | 312 | FBG | < 5.6 mmol/L<br>5.6-6.9 mmol/L | Mortality | ref<br>HR 4.11(1.15–14.74) [c] | [36] |
| Liu et al. | Single-center, retrospective study | Hospitalized | China | February 1–24 2020 | 255 | FPG | < 7 mmol/L<br>7–11.0 mmol/L<br>≥ 11.1 mmol/L | ICU admission | ref<br>HR 5.538(2.269–13.51) [d]<br>HR 11.55(4.45–29.99) [d] | [37] |

| | | | | | | | | | | |
|---|---|---|---|---|---|---|---|---|---|---|
| Wu et al. | Two-center, retrospective study | Hospitalized | China | December 26 2019–March 15 2020 | 2,041 | BG | ≥ 6.1 mmol/L | Progression to critical cases/death among non-critical cases<br>In-hospital mortality in critical cases | HR 1.30(1.03–1.63) [e]<br>HR 1.84(1.14–2.98) [e] | [38] |
| Liu et al. | Two-center, retrospective cohort study | Hospitalized | China | N.d. | 123 | FBG | ≥ 6.5 mmol/L | Critical illness ** | OR 1.249(1.032–1.521) | [39] |
| Fadini et al. | Retrospective observational study | Hospitalized | Italy | Feburary 21–April 20 2020 | 413 | FPG | Each 2 mmol/L increase | Severity | RR 1.21(1.11–1.32) | [40] |
| Smith et al. | Single-center, retrospective study | Hospitalized | USA | March 16–May 2 2020 | 184 | mean FBG | | Severity (requiring intubation) | Intubated: 238.0 mg/dL<br>Not intubated: 163.7 mg/dL | [41] |
| Zhang et al. | Single-center, retrospective cohort study | Hospitalized | China | February 8–March 21 2020 | 166 | FPG | < 7.0 mmol/L with no history of diabetes<br>≥ 7.0 mmol/L HbA1c < 6.5% Secondary hyperglycemia | ICU admittion, mechanical ventilation or death | ref<br>OR 2.61(0.86–7.88) [f] | [42] |
| Li et al. | Two-center, retrospective cohort study | Diabetes | China | December 31 2019–April 5 2020 | Total 132<br>T1D 2, T2D 130 | FBG | > 11 mmol/L<br>≤ 11 mmol/L | Death<br>In-hospital complications *** | OR 7.629(1.391–37.984)<br>OR 3.232(1.393–7.498) | [43] |
| Klonoff et al. | Multi-center retrospective cohort study | Admitted to ICU | USA | March 10–31 2020 | Total 1,544<br>ICU patients | BG | < 7.77 mmol/L<br>> 13.88 mmol/L | Mortality | ref<br>HR 3.14(1.44–6.88) [g] | [44] |

| | | | | | | | | | | | |
|---|---|---|---|---|---|---|---|---|---|---|---|
| Li et al. | Single-center, retrospective study | Hospitalized | China | January 22–March 17 2020 | 453 | Normal glucose | < 5.6% HbA1c | Mortality | HR 1.00 | | [45] |
| | | | | | | Hyperglycaemia | Fasting glucose 5.6–6.9 mmol/L and/or HbA1c 5.7–6.4% | | HR 3.29(0.65–16.6) | h | |
| | | | | | | Newly diagnosed DM | ≥ 7 mmol/L and/or HbA1c ≥ 6.5% | | HR 9.42(2.18–40.7) | h | |
| | | | | | | Known diabetes | | | HR 4.63 (1.02–21.0) | h | |
| Coppelli et al. | Single-center, retrospective study | Hospitalized | Italy | March 20–April 30 2020 | 271 | BG | ≥ 7.78 mmol/L with no history of diabetes | Mortality | HR 1.80(1.03–3.15) | a | [46] |

FPG: fasting plasma glucose, FBG: fasting blood glucose, T1D: type 1 diabetes, T2D: type 2 diabetes. * ARDS, acute cardiac injury, acute kidney injury, acute liver injury, cerebrovascular accident, coagulopathy, secondary infection. ** critical illness as a composite of admission to the intensive care unit (ICU), respiratory failure requiring mechanical ventilation, septic shock during hospitalization, or death. *** SARS-CoV-2-related ARDS, acute cardiac injury, acute kidney injury and secondary infection. [a] adjusted for sex and age. [b] adjusted for sex. [c] adjusted for age, sex, hospitals and comorbidities. [d] adjusted for age, gender, and disease duration. [e] adjusted for all available factors in baseline characteristics. [f] adjusted for age, sex, BMI, medical histories of hypertension, cardiovascular disease and malignancy. [g] adjusted for sex, age, baseline BMI, history of diabetes, and HbA1c. [h] adjusted for age, sex, smoking, systolic blood pressure and total cholesterol.

**Table S3.** Overview of the risk for adverse clinical outcomes of COVID-19 according to the glycemic control during hospitalization.

| Author | Study Population | | Region | Period | No. of Patients | Blood Glucose Level | | | Outcome | Risk | Ref. No. |
|---|---|---|---|---|---|---|---|---|---|---|---|
| | | | | | | | mmol/L | mg/dL | | | |
| Wu et al. | Two-center retrospective study | Critical cases | China | December 26 2019–March 15 2020 | Total 2041, non-critical 1690 at admission, critical 697 | median BG | ≥ 6.1 | ≥ 110 | Progression to critical cases/death among non-critical cases | HR 2.25 (1.78–2.84) * | [38] |
| | | | | | | | | | In-hospital mortality among critical cases | HR 2.39 (1.41–4.07) * | |
| Klonoff et al. | Multi-center retrospective cohort study | Non-ICU patients | USA | March 10–March 31 2020 | Total 1,544, non-ICU 1,184 | mean BG on day 2–3 | < 7.77 | < 140 | Mortality | ref | [44] |
| | | | | | | | > 13.88 | > 250 | | HR 7.17(2.62–19.62) # | |
| Bode et al. | Multi-center retrospective obsevational study | Hospitalized patients | USA | March 1–April 6 2020 | Total 1122, 184 in analysis | ≥2 BGs > 180 mg/ dL within 24 hour | | | Mortality | 41.7% vs 14.8% | [47] |
| | | | | | | with an HbA1c < 6.5% | | | Lengths of hospital stay | 6.2(1.1–20.7) days vs 5.0(1.1–24.6) days | |
| Zhu et al. | Multi-center retrospective cohort study | Hospitalized patients | China | December 30 2019–March 20 2020 | Total 7,337, Type 2 diabetes 952 (810 in analysis) | BG | 3.9–10.0 | 70–180 | Mortality | HR 0.14 (0.03–0.06) [&] [$] | [48] |
| | | | | | | | > 10.0 | > 180 | | ref | |

* adjusted for all available factors in baseline characteristics. # adjusted for sex, age, baseline BMI, history of diabetes, and admission BG. "[&] In the propensity score-matched model, age, gender, hospital sites, indicators of the severity of COVID-19, comorbidities (hypertension, coronary heart disease, cerebral vascular disease, chronic liver disease, and chronic renal diseases), and incidence of increased creatinine were matched". [$] Mixed-effect Cox model using the hospital site as a random effect and adjusting imbalanced durations from symptom onset to admission.
